# Role of Macrophages in the Repair Process during the Tissue Migrating and Resident Helminth Infections

**DOI:** 10.1155/2016/8634603

**Published:** 2016-08-25

**Authors:** Berenice Faz-López, Jorge Morales-Montor, Luis I. Terrazas

**Affiliations:** ^1^Unidad de Biomedicina, Facultad de Estudios Superiores Iztacala, Universidad Nacional Autónoma de México (UNAM), 54090 Tlalnepantla, MEX, Mexico; ^2^Departamento de Inmunología, Instituto de Investigaciones Biomédicas, UNAM, 04510 Ciudad de México, Mexico; ^3^Laboratorio Nacional en Salud, Facultad de Estudios Superiores Iztacala, UNAM, 54090 Tlalnepantla, MEX, Mexico

## Abstract

The Th1/Th2/Th17 balance is a fundamental feature in the regulation of the inflammatory microenvironment during helminth infections, and an imbalance in this paradigm greatly contributes to inflammatory disorders. In some cases of helminthiasis, an initial Th1 response could occur during the early phases of infection (acute), followed by a Th2 response that prevails in chronic infections. During the late phase of infection, alternatively activated macrophages (AAMs) are important to counteract the inflammation caused by the Th1/Th17 response and larval migration, limiting damage and repairing the tissue affected. Macrophages are the archetype of phagocytic cells, with the primary role of pathogen destruction and antigen presentation. Nevertheless, other subtypes of macrophages have been described with important roles in tissue repair and immune regulation. These types of macrophages challenge the classical view of macrophages activated by an inflammatory response. The role of these subtypes of macrophages during helminthiasis is a controversial topic in immunoparasitology. Here, we analyze some of the studies regarding the role of AAMs in tissue repair during the tissue migration of helminths.

## 1. Introduction

Helminth infections are a worldwide public health and economic problem due to their high morbidity rather than mortality. These infections are associated with socioeconomic (poor hygiene), demographic (living in endemic zones), health (obesity, diabetes, and viral infections such as human immunodeficiency virus (HIV)), and biological (raw meat consumption, sex, age, and immune response) factors, among others [1].

The clinical manifestations are diverse and include self-limited diarrhea, respiratory symptoms such as cough, wasting syndrome, and anemia. In severe infections, some people develop asthma-like symptoms [2] and neurologic disorders when the pathogen has the ability to migrate into the brain, such as in neurocysticercosis by* Taenia solium* [3, 4] and* Toxocara canis* infection [5], or motor disorders such as those occurring in* Trichinella spiralis* [6] and* T. canis* infections [7]. The diversity of symptoms caused by helminths is related to the organs they migrate to during their life cycle, such as the lung (*Ancylostoma duodenale*,* Ascaris lumbricoides*,* Strongyloides stercoralis*,* Brugia malayi*,* Dirofilaria immitis*,* T. canis*,* Schistosoma mansoni*,* Echinococcus granulosus*, and* Nippostrongylus brasiliensis*) [2], gall bladder and liver (*Schistosoma *sp.,* Toxocara *sp., and* A. lumbricoides*), muscle (*T. canis*,* T. spiralis*) [6, 7], and brain (*T. canis*,* Taenia *sp.) [3, 4].

In contrast to the prevailing consensus, many helminth infections are not permanent residents of the bowels; instead, they have the ability to migrate through different organs or persist at a location for weeks, months, or years. During their migration, helminths secrete proteolytic enzymes, such as serine proteases and cysteine proteases, with fibrinolytic effects that cause the disruption of cell junctions and the extracellular matrix, enabling entry to different organs [8–10] and causing tissue damage along the path of the migration.

## 2. Tissue Damage and Repair or Healing

**Figure 1:**
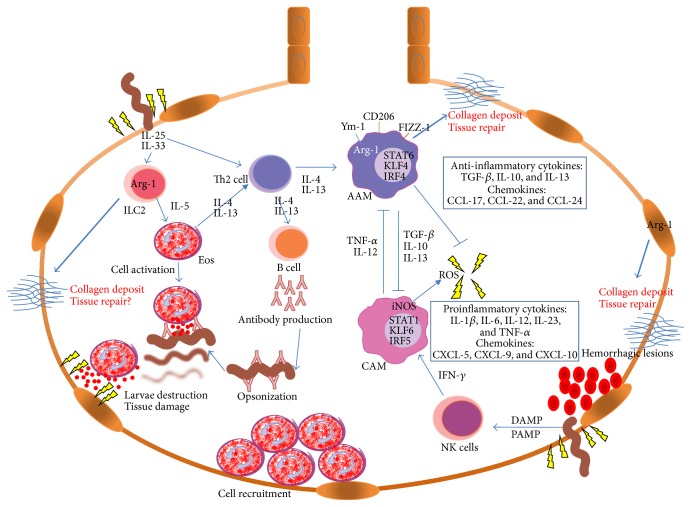
Immune response in tissue helminth infection. Lung tissue is usually affected during helminth migration; therefore, many repair mechanisms have been described for pulmonary tissue. The immune response is triggered when helminths disrupt the epithelial barrier. Helminths are a source of damage- and pathogen-associated molecular patterns (DAMPs and PAMPs), which activate various cells such as NK cells, epithelial cells, and innate lymphoid cells (ILCs). The production of IL-25 and IL-33 also activates ILCs, which are a main source of IL-5 that is important in eosinophil (Eos) activation. Eos bind antibodies linked to the parasite surface and release their intracytoplasmic enzymes during the acute phase of the infection, allowing parasite elimination; however, the surrounding tissue is also damaged. IL-25 and IL-33 also activate T helper lymphocytes type 2 (Th2), which in turn secretes IL-4 and IL-13, which promote B cell activation, antibody production, and the induction of alternative activated macrophages (AAMs). AAMs have two important mechanisms to decrease tissue damage. First, they inhibit the cytotoxic effect produced by classically activated macrophages (CAMs). Second, they produce enzymes, such as arginase-1 (Arg-1), that promote collagen production and deposition on damaged tissue, therefore restoring the function lost during CAMs and parasite-induced injury. AAMs also produce various cytokines (IL-10 and TGF-*β*) and chemokines (CCL-17, CCL-22, and CCL-24) and express markers such as YM-1, FIZZ-1, and MMR. On the other hand, CAMs are activated through IFN-*γ* production by natural killer (NK) cells and produce proinflammatory cytokines (IL-1*β*, IL-6, IL-12, IL-23, and TNF-*α*) and chemokines (CXCL-5, CXCL-9, and CXCL-10) and express iNOS that produces reactive oxygen species (ROS) and causes tissue damage. It is important to notice that although AAMs are fundamental in tissue repair, other cells such as ILCs and epithelial cells, which constitutively express Arg-1, may aid in tissue repair and produce collagen deposits in the damaged tissue.

When the larvae of helminths migrate through the host's body and destroy cells and tissues in their path, these cells and damaged tissue must be rapidly repaired. To achieve this, the host must turn on a series of coordinated biochemical and cellular events focused on the replacement of the destroyed tissue with living and functional tissue. Such a process is called tissue repair or tissue healing [11] and includes at least 4 events: bleeding, inflammation, proliferation, and remodeling (Figure 1) [12]. Bleeding and inflammation are early events that occur over hours or a few days, whereas proliferation and remodeling take weeks or even months. Vasculature changes such as vasodilatation and vasopermeability are rapidly induced to recruit cells to the site of damage, where prostaglandins and serotonin play an essential role. Once sufficient cell populations arrive at the site of the damaged tissue, the inflammatory stage ensues, with mast cells, neutrophils, and platelets as well as inflammatory Ly6C^hi^ monocytes and resident macrophages playing an important role in containing bleeding and initializing the first steps of bleeding control. Some of these cells (neutrophils and macrophages) have strong phagocytic activity and help to remove debris in the wound, an essential step in the repair process. This stage is mainly driven by macrophages and fibroblasts, which become activated and initiate the production or expression of different molecules such as platelet-derived growth factor (PDGF). PDGF induces fibroblast proliferation and promotes fibrogenesis, collagen precursors, and integrins that help in the communication among the extracellular matrix, inflammatory cells, fibroblasts, and parenchymal cells [12, 13]. Here, macrophages play a critical role as providers of many of the molecules necessary for tissue repair, including arginase-1, transforming growth factor- (TGF-) *β*, FIZZ-1 (found in inflammatory zone), and fibrin, which are involved in fibrosis by inducing myofibroblast differentiation, key elements in collagen (mainly type III collagen) and fibrin deposition [14]. Another cell population recently implicated with the repair processes in the lungs and intestines are the type 2 innate lymphoid cells (ILC2s), which are a source of cytokines and other components that participate in the early steps of the healing process [15]. This inflammatory stage must be mainly acute (1–4 days) to avoid excessive collagen accumulation and fibrosis, elements that may alter tissue architecture. Thus, these early events prepare the tissue for the next step in healing-proliferation. The proliferation of fibroblasts is a key event in this stage because they will migrate to the damaged area from similar neighboring tissue. Here, macrophages again play a primary role as providers of macrophage-derived growth factors (including fibroblast growth factor) that activate and induce proliferation in fibroblasts to provide new cells to repopulate the affected tissue [16]. The final and longest step in the repair and healing of the tissue consists of remodeling, which involves the reorganization of collagen fibers, which are “weak or fine” fibers occurring during the inflammation and proliferation stages. However, in the remodeling step, these fibers become strong because collagen type III is replaced with collagen type I, with more cross-links, as it enters into a reorientation process [14]. Thus, collagen synthesis continues during this final step to “mimic” the damaged tissue as much as possible indicating that tissue repair is a dynamic and long-lasting process.

## 3. Immune Activation by Helminths

In addition to proteolytic enzymes, helminths generate immunomodulatory antigens that induce predominantly Th2-biased responses. This type of immunity is characterized by the production of different immunoglobulin (Ig) subclasses, such as IgE, IgG1, and IgG4, as well as interleukins, IL-4, IL-13, IL-5, and IL-10, which benefit the expansion of diverse cellular subpopulations such as eosinophils, mast cells, helper T cells, and alternatively activated macrophages (AAMs). The interactions among these different cell types and antibodies promote allergy and hypersensitivity, which are related to the increase in vascular permeability, angiogenesis, cellular recruitment, smooth muscle contraction, mucus secretion by goblet cells, and collagen deposits, which together are important mechanisms of defense against invasive helminth infections [17].

Another cell population associated with these parasites is the CD4^+^Foxp3^+^ regulatory T cells (Tregs), which are essential to maintain immune homeostasis and prevent autoimmunity; however, they are also involved in the control of T_H_1 and T_H_2 immune responses in some infectious diseases. In fact, several reports have confirmed that an increase in Treg numbers is associated with different helminth infections, where they play a dual role. Such an increase in the regulatory cells appears to be permissive for parasites, whereas these high numbers of helminth-induced Foxp3^+^ Tregs dampen potentially pathogenic inflammatory responses or exacerbate Th2 responses in the tissue where the helminth has been allocated. Such conclusions were obtained by removing, stimulating, or transferring Foxp3^+^ Tregs using different experimental designs [18].

More recently, other cell populations were related to helminth infections, ILC2s. This cell population comprises a limited number of cells but appears to play an important role in both protective and repair processes in mucosal tissues. They are classified into two different populations: natural ILC2s (nILC2s) and inflammatory ILC2s (iILC2s). ILC2s have been found in the steady state in many organs, such as the lungs, liver, spleen, intestinal lamina propria, skin, bone marrow, and adipose tissue. These cells are stimulated by thymic stromal lymphopoietin (TSLP), IL-25, and IL-33 and after helminth infection produce effector cytokines, such as IL-4 and IL-13, and provide an important source of Th2-type cytokines. Thus, this process triggers the cell recruitment of eosinophils and macrophages, increasing the production of IL-13, IL-5, and IL-9, mucus production by goblet cells, muscle contraction, mastocytosis, tissue repair, and metabolic homeostasis [19]. The repair process by ILC2s is mostly associated with the early activation of AAMs, which can trigger the repair process via arginase-1 (Arg-1). However, a recent report has indicated that ILC2s can produce this enzyme both under basal conditions and in response to helminth infection; thus, ILC2s have the potential to participate in tissue repair [20].

During helminth infections, cytokines are essential to activate various cell types, including eosinophils, which are involved in the mediation of most helminth infections [21]. Eosinophils are mainly activated by IL-5 and are an important source of IL-4 and IL-13, which enable the activation of Th cells, AAMs, and mast cells. In addition to cytokine production and cell activation, eosinophils can neutralize and eliminate tissue parasites through the secretion of granules that contain proteins, such as eosinophil peroxidase (EPO), major basic protein (MBP), eosinophil derived neurotoxin (EDN), and eosinophil cationic protein (ECP) (Figure 1) [22].

These parasite-killing mechanisms have been described in multiple studies. For example, Masure et al. measured the survival of* Ascaris suum* second-stage larvae in the presence of eosinophils, and they observed an important reduction in larvae survival associated with the degranulation of eosinophils. The authors concluded that eosinophils are important immune cells in the defense against* A. suum* invasive larvae [23]. Other experimental models confirmed the protective role of eosinophils against multiple helminths, including* Strongyloides stercoralis* [24],* N. brasiliensis* [25], and* Heligmosomoides polygyrus* [26]. Nevertheless, during* T. canis* and* T. spiralis* infection, eosinophils do not seem to have such a protective role. For instance, in an* in vitro* model, Rockey et al. observed that eosinophils could attach to* T. canis* larvae and secrete granules; however, the larvae could separate from their sheaths and move away from eosinophils [4]. In another study, Takamoto et al. did not find a difference in the larvae burden of IL-5 deficient mice (characterized by having 3-fold lower circulating numbers of eosinophils), although eosinophils in WT mice were increased tenfold in the bone marrow and twenty-seven-fold in peripheral blood, and concluded that eosinophils do not play an important role in the clearance of* T. canis* larvae [27]. Another study using* T. spiralis* reported similar findings [28], suggesting that eosinophils do not enhance protective immunity also against this nematode.

Although it is clear that eosinophils play an important role in the protection against most helminths through the secretion of effector proteins, paradoxically, the proteins they release are sometimes harmful to the surrounding host tissues [29–32]. Evidence of this side effect was demonstrated in a model of* T. canis* infection, in which BALB/c mice were transfected with a plasmid encoding the IL-12 gene (pcDNA-IL-12) that inhibits the recruitment of eosinophils. The authors showed that transfected mice displayed reduced airway inflammation associated with a reduced eosinophilic infiltrate in the lungs and an increase in the Th1-type immune response characterized by elevated amounts of IL-12 and interferon-*γ* (IFN-*γ*) in this tissue [33]. In line with this idea, recently, a double-edged sword effect of eosinophils was demonstrated in experimental neurocysticercosis caused by* Mesocestoides corti*. Here, the authors used eosinophil-deficient mice to show that eosinophils are important cells for reducing the parasite load in the brain; however, these cells also intensify tissue damage and consequently worsen the disease outcome with more severe pathology [34].

Thus, after the damage to the host caused by helminth migration and immune cell activation, it is imperative that the tissue repair type 2 immune response plays a direct role in wound healing through the production of mediators that directly enhance the tissue repair process and through the control of inflammation, in which AAMs appear to have a central role [35]. However, a type 2 immune response takes several days; thus, a rapid mechanism for tissue repair is imperative early during the infection by helminths, when AAMs and their products appear to be crucial. In this regard, innate immune cells such as ILC2s may participate as an early source for IL-4 and IL-13 to rapidly induce AAMs.

## 4. Repair and Damage Mechanisms of Macrophages

Macrophage polarization to AAMs is related to the Th2 immune response and associated AAM cytokines, such as IL-4 and IL-13. Furthermore, diverse transcription factors, such as PU.1, signal transducer and activator of transcription 6 (STAT6), Kruppel-like factor (KLF) 4, and interferon regulatory factor (IRF) 4, are related to this type of macrophage [36]. Particularly, AAMs can be distinguished by their expression of diverse molecular markers, including the enzyme arginase-1 (Arg-1), members of the chitinase family (YM-1, YM-2, and AMCase), resistin-type molecules (FIZZ-1/Retnla/Relm-*α*, FIZZ-2/Retnlb/Relm-*β*, FIZZ-3/Retn/resistin, and FIZZ-4/Retnlg/Relm-*γ*), and TGF-*β* and mannose receptor (MMR/CD206) [37].

Moreover, classically activated macrophages (CAMs) are induced by Th1 immune responses, wherein IFN-*γ* plays a crucial role, and the transcription factors STAT1, KLF6, and IRF5 are implicated in their activation [36]. In contrast to AAMs, CAMs have enhanced antimicrobial actions mediated by the secretion of molecules such as nitric oxide (NO) and reactive oxygen species (ROS) that are essential for the destruction of intracellular pathogens (bacteria, viruses, and protozoan parasites). Additionally, CAMs are characterized by the production and secretion of proinflammatory cytokines, such as tumor necrosis factor- (TNF-) *α*, IL-12, and IL-1*β* [38]. Another difference between AAMs and CAMs is the expression by AAMs of Arg-1, which functions to metabolize L-arginine into L-ornithine. By contrast, CAMs use L-arginine to synthesize L-citrulline through inducible nitric oxide synthase (iNOS), producing NO and ROS, both mediators of their cytotoxic activity against intracellular pathogens and tissue damage [38].

## 5. Alternatively Activated Macrophage Functions

As previously mentioned, if macrophages are stimulated by IFN-*γ*, L-arginine is metabolized by iNOS, and the main cytokine induced is TNF-*α* or IL-12, macrophages will be classically activated. However, if there is predominance of Th2 cytokines, such as IL-4 and IL-13, there will be more AAMs that express Arg-1. Hence, AAMs expressing Arg-1 produce L-ornithine that can be metabolized into L-proline through ornithine aminotransferase (OAT), and L-proline is essential for collagen synthesis and tissue repair and regeneration [39]. AAMs also produce other elements involved in tissue repair, such as TGF-*β* and PDGF, which induce fibroblast proliferation and promote fibrogenesis and collagen production [40].

Another protein yielded by AAMs is YM-1, a member of the family of mammalian proteins that share homology to chitinases, which can bind chitin without chitinase activity. This protein has been associated with cellular recruitment and extracellular matrix deposition during tissue repair. Furthermore, FIZZ-1 is secreted in high amounts during inflammation, and it has been observed that diverse cells express this protein, including pneumocytes, alveolar epithelial cells, and macrophages. FIZZ-1 is also involved in fibrosis by inducing myofibroblast differentiation, key element in collagen and fibrin deposits [37, 41, 42]. Another immune factor related to AAM activation is antibody production. It has been described that some subclasses of antibodies can reprogram macrophage gene expression and induce the production of repair-related molecules [43].

## 6. Alternatively Activated Macrophages in the Repair Process in Diverse Pathologies

The presence of AAMs in the repair process has been extensively studied in various pathologies where they play an active role and could be beneficial or harmful depending on the pathology. In a collagenase-induced intracerebral hemorrhage (ICH) mouse model, an increasing number of CX3CR1 macrophages were revealed to have AAM markers. When these macrophages were depleted, an increase in the ICH lesion volume was observed, and neurological deficits were more severe compared to those of control mice, indicating a protective role of these macrophages in ICH. From such data, the authors concluded that brain-infiltrating macrophages after ICH are polarized to the AAM phenotype, thereby contributing to recovery from such injury [44].

In asthmatic airway inflammation, the presence of AAM activated via PU.1 has a harmful effect on promoting the pathological progress of asthmatic airway inflammation. Such an effect was measured in conditional PU.1-deficient (PU/ER(T)^+/−^) mice in response to the challenge of DRA (dust mite, ragweed, and* Aspergillus*) allergens, displaying attenuated allergic airway inflammation, decreased alveolar eosinophil infiltration, and reduced IgE production, changes associated with decreased mucus glands and goblet cell hyperplasia. To prove that AAMs were involved in the asthmatic airway pathology, macrophages from wild-type (WT) mice were differentiated with IL-4 and transferred to PU/ER(T)^+/−^ mice showing an increase in asthmatic airway inflammation. When the mice were treated with tamoxifen to rescue PU.1 function, the pathology was worsened compared with that in mice transferred with macrophages. The data indicated that PU.1 plays a critical role in AAM polarization and, indeed, in the exacerbation of asthmatic inflammation pathology [45].

Other pathologies such as obesity and resistance to insulin are closely associated with inflammation. Obesity causes increased classical and decreased alternative macrophage activation, which in turn induces insulin resistance in target organs, as observed in a study where A_2B_ adenosine receptor (AR) activation results in important regulators of macrophage activation. A_2B_ AR deletion results in impaired glucose and lipid metabolism associated with increased inflammatory classical macrophage activation and inhibition of anti-inflammatory alternative macrophage activation. The expression of AAM transcription factors was also decreased in the adipose tissue of A_2B_ ARs-deficient mice, indicating that AAMs may play an important role in obesity and insulin resistance, and therapeutic strategies targeting A_2B_ ARs could be a preventive therapy for those pathologies [46].

In a recent report, the role for AAMs has also been highlighted in the reparative processes after myocardial infarction in adult mice, where higher numbers of AAMs were recruited to the infarcted area; such mechanism was IL-4-dependent [47].

In general, in pathologies different from those generated by tissue migrating or resident helminths, the presence of AAMs in the repair process is beneficial to counteract the effects of CAMs associated with a Th1 inflammatory immune response, with the exception of asthma where their presence seems to be more harmful than beneficial.

## 7. Role of Macrophages during Tissue Migrating and Resident Helminths

### 7.1. *Nippostrongylus brasiliensis*

This nematode, like many others, has a tissue migration phase in which it migrates throughout the lungs, causing alveolar hemorrhage and inflammation. The role of the immune response during acute lung injury caused by* N. brasiliensis* has been studied (Table 1). In the experimental model using BALB/c mice, it was observed that IL-17 contributes to the inflammation. On the other hand, an increase in IL-4 receptor activation causes the reduction of IL-17 and enhances the expression of insulin-like growth factor-1 (IGF-1) and IL-10, with consequent AAM activation and tissue repair. These data highlight the essential role of Th2 cytokines and AAMs in limiting lung damage [48].

Another study using FIZZ/Retnla gene KO mice, a Th2-inducible gene, showed that there was greater lung and liver damage in Retnla^−/−^ mice infected with* N. brasiliensis* or* S. mansoni*, concomitant with exacerbation of fibrogenesis and increased IL-4 and IL-13 production, which in turn reduced parasite burden. These data suggest that the Retnla gene downregulates the Th2 immune response and suppresses resistance to nematode infection, granulomatous inflammation, and fibrosis [49].

The role of YM-1 during* N. brasiliensis* infection was also investigated. Sutherland et al., using neutralizing antibodies against YM-1 in an* in vivo* model, observed that YM-1 neutralization caused a decrease in neutrophils from bronchoalveolar lavage and lung tissue at 2 and 4 days after infection, followed by less inflammation but an increase in macrophages that was associated with lung healing [50]. Thus, even in the absence of YM-1, AAMs can still fulfill their repair function, and YM-1 appears to be implicated in neutrophilia and acute lung damage. However, other studies have confirmed that neutrophils are key elements in parasite neutralization and mediators of repair through AAM activation [51].

Other cells associated with tissue repair during* N. brasiliensis* infection include ILC2s, which are mainly induced by IL-25 and IL-33 [19] and to a lesser extent by IL-9, and appear to play an autocrine role amplifying ILC2s. The study was carried out on a model of IL-9R^−/−^ mice, in which IL-9R^−/−^ mice showed a significant decrease in ILC2s, IL-5, IL-13, and amphiregulin (a member protein of the epidermal growth factor family that promotes bronchoalveolar epithelium repair). Such a decrease was correlated with deficient tissue repair in the absence of IL-9. The tissue repair deficiency observed in this experimental setting was associated with a decrease in AAM markers, particularly Arg-1, Retnla, and YM-1, suggesting that ILC2s may induce AAMs to mediate the tissue repair process [52]. Nevertheless, there is recent evidence indicating that ILC2s constitutively express Arg-1 [20]. Moreover, Monticelli et al. demonstrated that ILC2s are the major source of Arg-1 in the lungs even more than alveolar macrophages in basal conditions; however, their role in lung inflammation is controversial [15].

### 7.2. *Toxocara canis*

Toxocariasis is a worldwide zoonotic parasitic disease caused by the nematode* T. canis*. In humans, the infection is caused by accidental ingestion of embryonated eggs from contaminated soil: the eggs hatch, and the liberated larvae migrate to different organs, producing various disorders. Murine models have shown the presence of transitory hemorrhagic pulmonary lesions associated with strong Th2 responses and heavy parasite burdens. During* T. canis* infection, there is predominance of a Th2 immune response, characterized by the production of IL-4, IL-5, IL-13, and immunoglobulin subclasses IgG1 and IgE, as well as an increase in peripheral blood eosinophils and eosinophilic granuloma in the lung and liver [53].

Although* T. canis* can migrate through diverse organs and the immune response described during this infection is prone to induce AAM, there are very few studies assessing their role in tissue repair. Only one study has evaluated the possible role of AAM markers during this infection. In this study, STAT6^−/−^ and WT mice were challenged orally with* T. canis* larvae, where longer persistence of hemorrhagic pulmonary lesions and inflammation in STAT6^−/−^ mice was observed to be associated with a weak Th2 immune response as a consequence of the inhibition of the STAT6 signaling pathway. By contrast, WT mice displayed strong Th2 immune responses, associated with high levels of IgG1, IgE, and IL-4 and the presence of AAM markers in lung tissue. Additionally, these WT mice resolved the lung lesions faster than STAT6^−/−^ mice. Interestingly, STAT6^−/−^ mice displayed significantly lower parasite loads. These data suggest that the severity in lung damage and persistence of lesions are associated with the absence of AAMs, as suggested for other helminth infections (Table 1) [54]. Strong inflammatory lung reactions in human toxocariasis have been well described, which may trigger chronic hypersensitivity mediated by an eosinophilic environment and granulomatous inflammation. Eosinophilic granuloma enables pathogen neutralization; however, it may have deleterious effects on the host, and AAM may play an important role in decreasing inflammation and favoring tissue repair, although its role in toxocariasis is yet to be determined [33, 55, 56]. Similarly, studies on the role of ILC2s in tissue repair during toxocariasis are lacking.

### 7.3. *Schistosoma mansoni*

AAMs are an essential cell type during schistosomiasis (Table 1), which are involved in the reduction of tissue inflammation and associated injury triggered by* S. mansoni* eggs deposited in liver tissue. To prove the role of this cell type, Herbert et al. used an experimental model of LysM^cre^IL-4^−/flox^ and IL-4 deficient mice, both with impaired activation of AAMs and with the enhanced ability to induce CAM expressing iNOS2. The authors also observed that WT mice had smaller liver granulomas and higher expression of Arg-1 than LysM^cre^IL-4^−/flox^ and IL-4 deficient mice [57]. Similar results were published by Vannella et al. using IL-4R*α* ^flox/Δ^LysM^Cre^ mice, concluding that AAMs are necessary to suppress pathogenic Th1/CAM responses without a significant impact on fibrosis, although fibrosis was slightly higher in IL-4R*α* ^flox/Δ^LysM^Cre^ mice [58].

By contrast, previous studies have shown that IL-4 and IL-13 (Th2-type response) may play dual roles in lung granuloma formation, which is necessary for* S. mansoni* egg containment, suggesting that Th2 immune responses may produce tissue damage rather than repair. For example, IL-13 induces tissue eosinophilia and high levels of IgE enhancing lung granuloma formation, whereas IL-13 blockage in mice was accompanied by changes in eosinophil accumulation and reduced granuloma size [59]. This is in line with another study that has established the fact that IL-13 exhibits chemotactic activity for human eosinophils; therefore, schistosome granulomas are rich in eosinophils [60]. Thus, damaged tissue is associated with eosinophil recruitment without participation of AAM, although both cell types are part of the Th2 immune response [59]. However, it has been recently observed that AAMs are also important in maintaining local Th2 responses in general and IL-13 production in particular during* S. mansoni*-induced granuloma formation, as demonstrated by partial AMM depletion that overall reduces lung fibrosis and pulmonary inflammation, as described by Borthwick et al. [61].

In an* in vitro* study, using bone marrow macrophages differentiated with IL-10 and IL-4 to AAMs, upregulation of FHL2 (a protein structural domain, also called LIM) was observed. However, when the bone marrow macrophages from FHL2^−/−^ mice were similarly stimulated, the AAM genes were downregulated, and CAM markers seemed to be upregulated, proving the expression of FHL2 induced in mouse marrow-derived macrophages following stimulation with AAM-inducer cytokines. To prove the importance of FHL2 in AAM activation, FHL2^−/−^ mice were challenged with* S. mansoni* showing higher numbers of granulomas and reduced expression of AAM markers, which correlate with an enhanced Th1 immune response. These data suggest a role for FHL2 in the pathogenesis of pulmonary granulomatous inflammation through AAM polarization and Th1/Th2 balance [62].

During intestinal schistosomiasis by* S. mansoni*, the use of* S*-(2-boronoethyl)-L-cysteine (BEC), an Arg-1, and Arg-2 antagonist, was related to impaired elimination of* S. mansoni* eggs that correlated with an increase in disease severity and mortality compared with that in nontreated mice. In the same study, now using Arg-1^−/−^ mice, the authors observed hemorrhagic lesions in the intestinal mucosa that were not observed in WT mice. These data confirmed that Arg-1 production by AAMs is important for both* S. mansoni* infection control and reducing intestinal damage, and the absence of Arg-1 causes Th1 polarization associated with a proinflammatory cytokine profile [63].

Evidence that a decreased Th1 immune response and a reduced number of CAMs and therefore indirect stimulation of AAM contribute to repair mechanisms has been shown in CD14 (a TLR-4 coreceptor) deficient mice. These mice have fewer and smaller hepatic granulomas and an increase in CD4^+^IL-4, IL-5, IL-13^+^, and CD4^+^Foxp3^+^IL-10^+^ cells that correlate with collagen deposition and wound healing. This effect was associated with STAT6 signaling, suggesting that the absence of CD14 has an impact on the ILR4*α*/STAT6 pathway and macrophage polarization during infection [64, 65]. Moreover, the inhibition of cytokines or factors related to AAM polarization causes a decrease in the protective role of AMM. In conclusion, most data point out these cell types as vital for the successful repair of lesions during schistosomiasis [66, 67].

### 7.4. *Heligmosomoides polygyrus bakeri*

As mentioned above, antibodies also mediate AAM activation. This fact has been observed during* H. polygyrus bakeri* infection (Table 1). Esser-von Bieren et al. described that complement-dependent antibodies that bind to FcR*γ* cause macrophage adherence to* H. polygyrus* larvae* in vitro*, immobilizing the parasite, triggering macrophage reprogramming, and enhancing the expression of genes associated with repairing mechanisms. Such mechanisms were independent of the IL-4R*α* signaling pathway, suggesting a different AAM activation mechanism [43].

Other studies have proven that cardiac resident macrophages, in the absence of infection, expressed classical (IL-1*β*, TNF-*α*, and CCR2) and alternative (Ym-1, Arg-1, RELM-*α*, and IL-10) markers. Nevertheless, during* H. polygyrus* infection, there is an increase in the expression of Ym-1, RELM-*α*, and CD206 and enhanced collagen deposition, causing fibrosis in heart tissue. Although* H. polygyrus* is a local migratory tissue parasite (which penetrates submucosal layer of the small intestine to the muscularis externa and later towards the lumen), polarization of AAM is induced by the immunologic activation of infection [68]. However, there is a lack of information regarding the role of AAMs in cardiac tissue repair. Although it has been suggested that fibrosis is a mechanism of tissue repair, it is still necessary to determine the positive or negative fibrotic effect on the heart during* H. polygyrus* infection. However, the idea that an intestinal helminth infection could have an effect on an organ as the heart is very interesting to explore.

### 7.5. *Trichinella spiralis*

During trichinellosis by* T. spiralis*, it has been reported that macrophages are important mediators of inflammation in adipose tissue, insulin resistance, and glucose control (Table 1). Therefore, the role of AAMs in obesity has been studied in the context of helminth infection. Using an experimental model of obese mice infected with* T. spiralis*, the induction of AAMs triggered by helminth infection led to decreased glucose intolerance and consequent lowering of the blood glucose levels which was associated with AAM markers such as Arg-1, CD206, and IL-10, as well as adipocyte death [69]. These results suggest that AAMs, which are induced by* T. spiralis* infection, have a beneficial role during obesity through the regulation of the inflammatory process in adipose tissue.

In other inflammatory diseases, such as colitis (Table 1), it has been observed that excretory/secretory (ES) proteins produced by these parasites have immunomodulatory effects. Among those ES proteins produced by* T. spiralis*, the recombinant 53 kDa protein rTsP53 was found to polarize the immune response to the Th2 phenotype. Using this protein during experimental colitis caused a Th2 immune response that correlates to reduced inflammation and the enhanced expression of the AAM markers Arg-1, FIZZ-1, TGF-*β*, and IL-10 [70]. These anti-inflammatory effects triggered by rTsP53 appear to be helpful in repairing tissue during colitis.

### 7.6. *Taenia crassiceps*

*T. crassiceps* is a cestode that has been extensively studied. These parasites induce a population of AAMs with suppressive activity and have been shown to have immunomodulatory effects on experimental colitis and colon cancer models (Table 1) [71, 72]. In this regard, AAMs play a central role in modulating both colonic inflammation and colitis-associated tumorigenesis. During experimental colitis, it has been shown that* T. crassiceps* infection induces the expression of Arg-1, YM-1, and FIZZ-1, which is related to increased collagen deposition in the intestine that does not cause fibrosis but diminishes intestinal inflammation and hemorrhage. Moreover, when AAMs isolated from* T. crassiceps*-infected mice were adoptively transferred to colitic mice, these cells could ameliorate ongoing colitis [71, 72].

A similar effect was observed in experimental autoimmune encephalomyelitis (EAE), in which the presence of* T. crassiceps* causes a decrease in inflammation and symptoms of encephalomyelitis with repair in the spinal cord. Such effects were associated with anti-inflammatory cytokine production and expression of AAM markers [73]; together, these data indicate that sometimes helminth infections generate improved side effects.

### 7.7. *Trichuris muris*

There is limited information regarding the role of AAMs during infection of the intestinal nematode* Trichuris muris *(Table 1), but it has been established that it induces a Th2 immune response and therefore induction of AAMs. The only study that has investigated the role of AAM during* T. muris* infection showed that Arg-1 is dispensable for tissue repair, and its absence was not related to more damage [74]. However, it was not determined whether other mechanisms associated with AAM or not were responsible for tissue healing.

## 8. Concluding Remarks

The type 2 immune response has evolved to direct the wound-healing machinery not only to repair and remodel tissue but also to mediate the containment, destruction, or expulsion of helminths. Both effects have been associated with the presence of AAMs; particularly, the issue related to tissue repair has also been related to other mechanisms independent of AAMs, such as ILC2s and epithelial cells from lung tissue that constitutively express Arg-1 for collagen production. Consequently, those cells could also be involved in tissue repair. Given such information, another way to prove the importance of AAMs and other sources of collagen production in the repair process during helminth tissue migration could be depleting macrophages from lung tissue and measuring Arg-1 and collagen deposition associated with ILC2s or pulmonary epithelial cells. Even though there has been great progress in the understanding of AAMs functions and their role in tissue repair, a full depletion of AAMs from lung tissue has not been achieved, and there are still points of uncertainty and controversies that must be resolved in the future.

## Figures and Tables

**Table 1:** Alternatively activated macrophages (AAMs) in the repair process during tissue migration and resident helminthes.

Helminth	Experimental model and mice strain	AAMs role	Ref.
*Nippostrongylus brasiliensis*	BALB/c	IGF-1 production is increased by IL-4, inducing AAM activation followed by tissue repair	[48]
	FIZZ/Retnla^−/−^	The Retnla gene downregulates the Th2 immune response and suppresses resistance to nematode infection, granulomatous inflammation, and fibrosis	[49]
	BALB/c	In the absence of YM-1, AAMs can still fulfill their repair function	[50]
	BALB/c	Neutrophils are key elements in parasite neutralization and are mediators of repair through AAM activation	[51]
	IL-9^−/−^	ILC2s may induce AAMs, which mediate adequate tissue repair	[52]
	STAT6^−/−^ and Arg-1^flox^	ILC2s constitutively express Arg-1, and they can repair lung tissue during acute inflammation in the absence of AAMs	[20]

*Toxocara canis*	STAT6^−/−^	STAT6 absence may cause delayed wound healing by the reduction of the AAM population	[54]

*Schistosoma mansoni*	LysM^cre^IL-4^−/flox^ and IL-4^−/−^	Polarization to the Th1 immune response, associated with CAM activation and NOS production, is related to hepatic damage and death	[57]
	IL-4R*α* ^flox/Δ^LysM^Cre^	AAMs are necessary for pathogenic Th1/CAM suppression	[58]
	FLH^−/−^	The absence of FHL induces Th1 polarization, which is associated with an increase in granuloma hepatic formation	[62]
	Arg-1^−/−^	Arg-1 production by AAMs may play an important role in *S. mansoni* infection control and diminish intestinal damage	[63]

*Heligmosomoides polygyrus bakeri*	J_H_^−/−^ and FcR*γ* ^−/−^	Antibodies cause AAM activation, enhancing the expression of genes associated with the repair mechanisms	[43]
	C57BL/6 and Cx3Cr1^GFP/+^	Infection increases the expression of Ym-1, RELM-*α*, and CD206, enhancing collagen deposition and fibrosis in heart tissue	[68]

*Trichinella spiralis*	Ob/ob and C57BL/6	AAMs mediate inflammation in adipose tissue, insulin resistance, and glucose control	[69]
	BALB/c	The rTsP53 protein appears to be beneficial during colitis, with an increase in AAMs	[70]

*Taenia crassiceps*	BALB/c	AAMs have immunomodulatory effects in experimental colitis and colon cancer models	[71, 72]
	C57BL/6	A decrease in inflammation and EAE symptoms is associated with AAM activation	[73]

*Trichuris muris*	Arg-1^flox/flox^, Tie2-cre, and C57BL/6	Arg-1 is dispensable for tissue repair, but its absence is not related to damage	[74]
